# Changes in Nucleus Pulposus Cell Atlas and the Role of SPP1 During Intervertebral Disc Degeneration: Single-Cell Sequencing Analysis

**DOI:** 10.1155/mi/5593429

**Published:** 2025-11-29

**Authors:** Chen Liu, Kun Jiao, Xiaoyu Li, Zixiang Deng, Shanhe Wang, Yajun Cheng, Ming Li, Xiaoyi Zhou, Xianzhao Wei

**Affiliations:** ^1^Department of Orthopaedic Surgery, The First Affiliated Hospital of Naval Military Medical University, Shanghai 200433, China; ^2^Fifth Students Brigade, The Basic Medical University of Naval Military Medical University, Shanghai 200433, China

**Keywords:** IVDD, nucleus pulposus, scRNA-seq, SPP1

## Abstract

**Background:**

The nucleus pulposus (NP) plays a central role in the pathogenesis of intervertebral disc degeneration (IVDD); however, its internal cellular heterogeneity and molecular mechanisms have not yet been elucidated.

**Methods:**

ScRNA-seq was used to evaluate the structure of the NP at different degenerative stages in the same individual with IVDD. Unsupervised clustering of cells based on gene expression profiles was performed using the Seurat package and passed to uniform manifold approximation and projection (UMAP) for cluster visualization. A rat disc degeneration model and an in vitro human NP cell degeneration model were established to validate the scRNA-seq identification results.

**Results:**

Six NP subclusters and immune cells were identified and their distribution and functional differences between healthy and degenerative states were investigated. Immune cells were present only in degenerated NPs and may trigger NP degeneration. Cellular communication within the NP was altered by the intervention of immune cells. Secreted phosphorylated protein 1 (SPP1), secreted by immune cells, plays a major role and is a key molecule in NP degeneration. The results of in vivo animal experiments and in vitro cellular experiments showed that the expression of SPP1 was increased in degenerating NPs. High expression of SPP1 promoted NP degeneration, whereas inhibition of its expression attenuated degeneration.

**Conclusions:**

Cytoarchitectural changes in NP were revealed by scRNA-seq. SPP1 is involved in the pathogenesis of disc degeneration and may be a new target for intervention in IVDD.

## 1. Background

Low back pain (LBP) is a leading disabling health condition in humans, with a lifetime prevalence reaching up to 84% [1]. Intervertebral disc degeneration (IVDD) is a widely recognized contributor to LBP [2]. The current treatment of IVDD, mainly including bed rest, rehabilitation, medication, interventional therapy, and surgery [3], provides only symptomatic relief but fails to reestablish homeostasis of the IVDs. An increased understanding of human IVD physiology and pathology is necessary.

Mature IVDs consist of a central nucleus pulposus (NP), surrounding annulus fibrosus (AF), and cartilage endplate (CEP), which adjoins the vertebra [4]. The origin of the IVD is heterologous; the NP is believed to be derived from the notochord, and the AF and CEP from the sclerotome [5]. The NP is rich in collagen type II (COL2) and proteoglycans. They facilitate osmotic properties and allow the retention of the fluid required to maintain the NP height and turgor against compressive loads [6]. Thus, it has been widely studied in IVDD.

Current studies on the pathophysiology of NP are supported by transcriptomic and epigenomic analyses. However, the development and application of single-cell sequencing technology can help explore the nature of the disease, reveal changes in NP cell types and intercellular communication during degeneration, and provide new ideas for the treatment of disc degeneration.

In this study, we profiled 7633 cells from the NP of the same individual at different stages of degeneration. By analyzing single-cell sequencing data, we explored the cellular heterogeneity within the NP before and after degeneration and found that infiltration of immune cells into the interior of the NP affects intercellular crosstalk, with an important role played by the secreted phosphorylated protein 1 (SPP1) signaling pathway. Our results provide new cellular-level insights into the transcriptional alterations associated with IVDD, which could be used in the development of preventative and regenerative strategies for IVDD.

## 2. Materials and Methods

### 2.1. Single-Cell RNA-Seq Data Analysis

#### 2.1.1. Single-Cell RNA-Seq Data Processing

Unbiased transcriptome-wide scRNA-seq and computational analyses were performed and raw sequencing data for each sample were converted to matrices of expression counts using the Cell Ranger software 10x Chromium Single Cell 3 provided by 10x Genomics. In brief, raw BCL files from the Illumina HiSeq4000 were demultiplexed into paired-end GZIP-compressed FASTQ files using Cell Ranger's mkfastq. Using Cell Ranger's count, reads were aligned to the GRCh38 human reference genome and transcript counts were quantified for each annotated gene within each cell [7]. The resulting UMI count matrices (genes × cells) were provided as inputs to Seurat Suite (version 4.3.1) [8]. Expression matrix files for subsequent analyses were generated based on the gene and UMI counts. Cells were filtered using gene counts between 200 and 6000 and UMI counts below 50,000. Cells with more than 20% mitochondrial content were excluded. Seurat functions were used for dimension reduction and clustering. All gene expression levels were normalized and scaled using NormalizeData and ScaleData.

#### 2.1.2. Dimension Reduction and Clustering

We used principal component analysis (PCA) to analyze the top 2,000 variance genes, which were selected using FindVariableFeatures [9]. Clustering and visualization of the integrated data set were performed using uniform manifold approximation and projection (UMAP), an unsupervised nonlinear dimensionality reduction technique, based on the first 20 principal components with a resolution of 0.4 (FindClusters and RunUMAP functions in Seurat).

#### 2.1.3. Cell Cluster Annotation

We calculated the marker genes using the FindAllMarkers function with the Wilcox rank-sum test algorithm under the following criteria: (1) ln FC > 1, (2) adjusted *p* value < 0.05, and (3) min. pct > 0.01. Then, we identified the cell types and matched the marker genes of the corresponding cluster to specific cell types based on the “SingleR” [10] package and the CellMarker database [11].

#### 2.1.4. GSEA

Fifty classical gene sets downloaded from the GSEA website (GSEA|MSigDB [gsea-msigdb.org]) were used as references to further understand the biological functions of differentially expressed genes in different cell subtypes. Pathway analysis was used to identify significant pathways of marker genes and differentially expressed genes based on the KEGG database. Fisher's exact test was used to select significant pathways and the threshold of significance was defined by the *p* value and FDR.

#### 2.1.5. Pseudotime Analysis

Single-cell trajectory analysis was performed using Monocle2 [12] (version 2.28.0; http://cole-trapnell-lab.github.io/monocle-release) to reveal cell state transitions in the NP and immune cell clusters. Dimensional reduction and cell ordering were performed using the DDRTree method and the orderCells function. Before monocle analysis, marker genes of the Seurat clustering results and raw expression counts of the filtered cells were selected.

#### 2.1.6. Cell Communication Analysis

CellChat [13] (version 1.6.1) analysis was performed to assess cell-to-cell communication in whole-cell populations. CellChat is a tool for inferring and analyzing intercellular communication networks using network analysis and pattern recognition methods to predict the major signaling inputs and outputs of cells and how these cells and signals coordinate their functions. CellChat assesses the impact of intercellular interactions based on intercellular ligand and receptor expression. We focused on the apparent differences in cellular ligand–receptor interactions during cellular communication between degenerated and healthy NP samples to explore the important role of cellular ligand–receptor interactions in IVDD. Significant means and cell communication significance (*p*  < 0.05) were calculated based on the interaction and normalized cell matrix achieved by Seurat Normalization.

### 2.2. Rats and Treatment

A rat model of IVDD was generated by performing surgery under aseptic conditions. In brief, the rats were placed in the prone position, the entire tail was shaved and cleaned (70% ethanol dissolved in double-distilled water), and a 1–1.5 cm longitudinal incision was made centered on the caudal vertebrae 6 and 7, and the skin was incised to expose the location of the intervertebral disc. Puncture with a 20-g needle was used to simulate disc degeneration in the rat caudal spine and the skin was closed with a 4-0 silk suture. The control group did not undergo puncture. All animal experiments were conducted according to the guidelines approved by the Institutional Animal Care and Use Committee at the Navy Medical University.

### 2.3. Magnetic Resonance Imaging (MRI)

Two weeks after surgery, MRI was performed on all rats before sacrifice. After anesthetization, the rats were placed in a prone position with their spines straight. The degree of degeneration observed on MRI was determined according to the Pfirrmann grade by two spine surgeons.

### 2.4. X-Ray

Two weeks after surgery, X-ray was performed on all rats before sacrifice. After anesthetization, the rats were placed in a prone position with their spines straight. Two spine surgeons observe of intervertebral space height to determine disc degeneration.

### 2.5. Immunohistochemistry (IHC) and Histopathological Analysis

Tissue specimens were embedded in paraffin and cut into 5-µm sections. Subsequently, the sections were deparaffinized and rehydrated, followed by hematoxylin and eosin (H&E) and Safranin-O (S-O) staining, or antigen retrieval with 0.01 M sodium citrate. Sections were blocked with 3% hydrogen peroxide and 5% normal goat serum. The slides were then incubated with primary antibodies, included anti-spp1 (30200-1-AP; Wuhan Sanying). The sections were incubated with a secondary antibody and developed using DAB solution. H&E was used for nuclear and cytoplasmic staining. Finally, the sections were observed and imaged under an Olympus BX63 microscope and a polarized microscope (Leica) at ×10, ×50, and ×400 magnification; and the expression of SPP1^+^ cells in the IVD samples was quantified using ImageJ software (National Institutes of Health). Histological scores were assigned to the normal and degenerated discs.

### 2.6. NP Cell Culture

The cells were cultured in complete medium for immortalized NP cells (QuiCell) at 37°C in 5% CO_2_. The medium was replaced twice weekly. NP cells were inoculated into six-well plates and cultured to 80% confluence for subsequent experiments.

### 2.7. In Vitro siRNA Transfection

Small interfering RNAs (Human SPP1 siRNA, targeting sequences #1 GTCTCACCATTCTGATGATGAA, #2 GAACGACTGATGATGTA, and #3 CCAAGTAAGTCCAACGAAA) were constructed by RiboBio and used to inhibit the expression of SPP1. NP cells were cultured in six-well plates to 60%–70% confluence and transfected with negative control or SPP1 siRNA using Lipofectamine 3000 (Thermo Fisher Scientific) according to the manufacturer's instructions. After 48 h, cellular lysates were obtained to analyze the expression of the genes of interest.

### 2.8. Real-Time Polymerase Chain Reaction (RT-qPCR)

Total RNA was isolated from NP tissues or cultured cells using the TRIzol reagent (TaKaRa Bio) according to the manufacturer's instructions. The RNA quantity was analyzed using a NanoDrop spectrophotometer (Thermo Fisher Scientific). mRNA was converted to cDNA using Prime Script RT Master Mix (TaKaRa). All reactions were run on a RT-PCR system (Applied Biosystems) and analyzed using the comparative Ct (ΔΔCt) method (2^-ΔΔCt^ with logarithmic transformation). The following primers were used: human SPP1 (F: 5′-CTCCATTGACTCGAACGACTC-3′, R: 5′-CAGGTCTGCGAAACTTCTTAGAT-3′); human GAPDH (F: 5′-AATGGACAACTGGTCGTGGAC-3′, R: 5′-CCCTCCAGGGGATCTGTTTG-3′).

### 2.9. Western Blot (WB) Analysis

The proteins of the treated NP cells were extracted and electrophoretically separated using 10% or 15% sodium dodecyl sulfate-polyacrylamide gel electrophoresis. Subsequently, the membranes were blocked with 3% bovine serum albumin (BSA) and incubated with primary antibodies. The primary antibodies included anti-SPP1 (30200-1-AP; Wuhan Sanying), anti-aggrecan (13880-1-AP; Wuhan Sanying), and anti-MMP3 (17873-1-AP; Wuhan Sanying). After washing with PBS, membranes were incubated with anti-rabbit IgG (7074; Cell Signaling Technology) or anti-mouse IgG (7076; Cell Signaling Technology) antibodies. Finally, the membrane was pressed in a dark room, exposed, and analyzed.

### 2.10. Toluidine Blue Staining and Immunofluorescence (IF)

NP cells were grown on confocal plates, incubated for 24 h, and then treated as needed for 24 h. The cells were washed three times with PBS buffer precooled to 4°C, followed by fixation with 1 mL of 4% paraformaldehyde solution per well at room temperature for 20 min. After fixation, the cells were washed three times with PBS buffer precooled to 4°C and then stained with 1 mL of toluidine blue staining solution per well.

The cells were fixed for 15 min with 4% formalin and permeabilized for 10 min with 0.1% Triton X-100. After washing, cells were blocked for 1 h with 10% goat serum, incubated with diluted anti-SPP1 (30200-1-AP; Wuhan Sanying), antiaggrecan (13880-1-AP; Wuhan Sanying), and fluorescent secondary antibody, and observed under a fluorescence microscope.

## 3. Results

### 3.1. Identification and Occupancy of IVDD NP Cell Subpopulations

**Figure 1:**
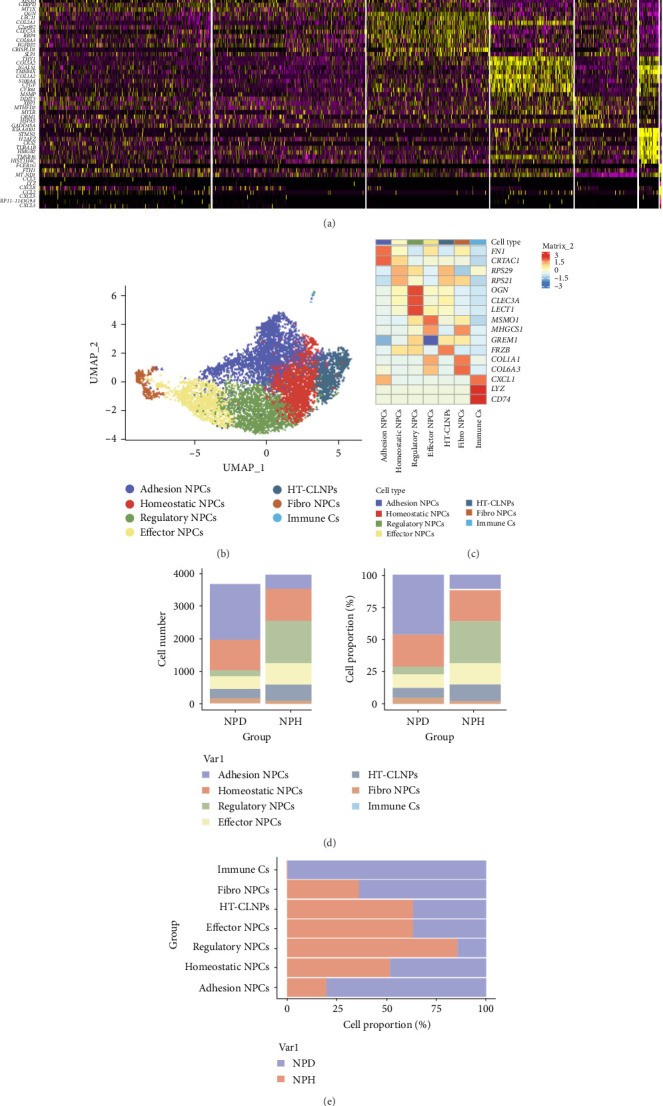
Identification of seven cell clusters with diverse annotations revealing high cellular heterogeneity in NP based on single-cell RNA-seq data. (a) Heatmap illustrates the top 10 differential genes in each cell cluster. (b) All seven cell clusters in NP were annotated with singleR and CellMarker according to the composition of marker genes. (c) Expression levels of marker genes for each cell cluster. (d, e) Number and proportion of each cell cluster before and after degeneration.

We analyzed scRNA sequencing data from the GSE199866 dataset, which included one healthy NP tissue and one degenerative NP tissue from the same person. After quality control and doublet exclusion filtering to remove cells with low gene detection (<200 genes) and high mitochondrial gene content (>20%; Figure S1A), 7633 cells from NP tissues were included in the study to provide a single-cell view of IVDD pathology. Using variance analysis, we acquired the top 2000 highly variable genes (Figure S1B). Next, we performed PCA to reduce the dimensions of the data. Subsequently, the UMAP algorithm was used to cluster the 20 principal components and all cells were classified into seven cell clusters (Figure S1C). A heatmap illustrates the top 10 differential genes in each cell cluster (Figure 1a). Cell clusters were annotated according to the cell marker database and published IVDD single-cell studies (Figure 1b).

NP cells were identified based on the levels of transcripts encoding different proteins (e.g., aggrecan proteoglycan [ACAN] and SRY-box transcription factor 9 [SOX9]) [14] (Figure S1D). In addition to NP cells, immune cells were identified in NP tissues (Cluster 6; expressing *LZY*, *CXCL1*, and *CD74*) [15, 16] (Figure 1c). Six subpopulations were identified based on highly expressed genes and published single-cell data (Figure 1c):

1. Adhesion NP cells (Cluster 1: mRNAs related to cell adhesion and migration such as *FN1* [17] and *CRTAC1* [18]);
2. Homeostatic NP cells (Cluster 2; expressing *RPS29* and *RPS21*) [19];
3. Regulatory NP cells (Cluster 3: *OGN* [20], *CLEC3A* [21], and *LECT1* [22]);
4. Effector NP cells (Cluster 4, expressing mRNAs that encode proteins that participate in cellular metabolic genes, e.g., *MSMO* [23] and *HMGCS1* [24]);
5. Hypertrophic chondrocyte-like NP cells (HT-CLNPs; Cluster 5, expressing *FRZB* [25]); and
6. Fibro-NP cells (Cluster 6, expressing mRNAs that encode proteins related to fibrosis, *COL1A1* and *COL6A* [19, 26]).

Bar graphs show the number and percentage of all cell types in different NP samples (Figure 1d,e). We found that immune cells were only present in degenerated NP tissues, and adhesion NP cells significantly increased in number and percentage as NP tissues degenerated and were the predominant cell type in degenerated NP tissues. In contrast, regulatory NP cells were the most common cell type in normal NP tissues.

### 3.2. Functional Enrichment Analysis and Pseudotime Analysis

**Figure 2:**
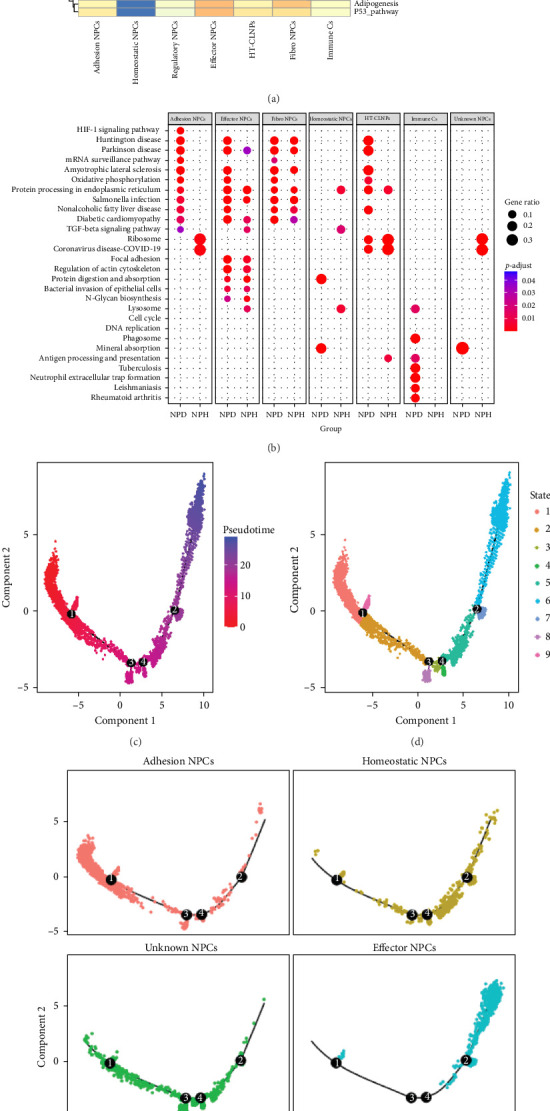
Functional enrichment analysis and pseudotime analysis of marker genes based on seven cell clusters. (a) Based on the marker genes of differentially expressed cells, ssGSEA for 50 HALLMARK functional enrichment. (b) Based on the marker genes of differentially expressed cells, ClusterProfiler package for KEGG functional enrichment. (c–e) Trajectory analysis revealed the evolution of NP cell subpopulation differentiation.

The biological functions of each cell subtype were analyzed using the ssGSEA algorithm with reference to a gene set of 50 HALLMARK biological pathways (Figure 2a). Adhesion NP cells were mainly enriched in the TNFA_SIGNALING_VIA_NFKB signaling pathway, which is involved in the inflammatory response; regulatory NP cells were significantly enriched in the KRAS_ SIGNALING_DN pathway; effector NP cells were enriched in the MYOGENESIS and HEDGEHOG_SIGNALING pathways; fibro-NP cells were mainly enriched in the SPERMATOGENESIS and E2F_TARGETS pathways. Immune cells were significantly enriched in the ALLOGRAFTREJECTION and IL6_JAK_STAT3_SIGNALING signaling pathways. In contrast, homeostatic NP cells and HT-CLNPs were not significantly enriched in any classical pathway.

KEGG pathway enrichment showed that adhesion NP cells in degenerated tissues were enriched in the HIF-1 signaling pathway compared with those in healthy NP tissues and that effector NP and fibro-NP cells were functionally similar and were enriched in cytoskeletal pathways, such as focal adhesion and regulation of the actin cytoskeleton. Homeostatic and regulatory NP cells were significantly enriched in mineral absorption pathways after degeneration (Figure 2b).

To study differentiation and corresponding gene expression in the different subpopulations, we selected all NP cell subpopulations and constructed a differentiation trajectory containing nine cell states (Figure 2c, d). Most of the adhesion NP cells appeared in State 1, which is the beginning of the entire pseudotemporal differentiation, and the effector NP cells appeared mainly in States 6 and 7, which are the entire pseudotemporal differentiation at the end of pseudotemporal discretization. In addition, fibro-NP cells showed a bipolar distribution in the differentiation trajectory, with a small fraction appearing at the beginning and most appearing at the end of the trajectory (Figure 2e).

### 3.3. CellChat Analyses Show Cell-to-Cell Communication in NP

**Figure 3:**
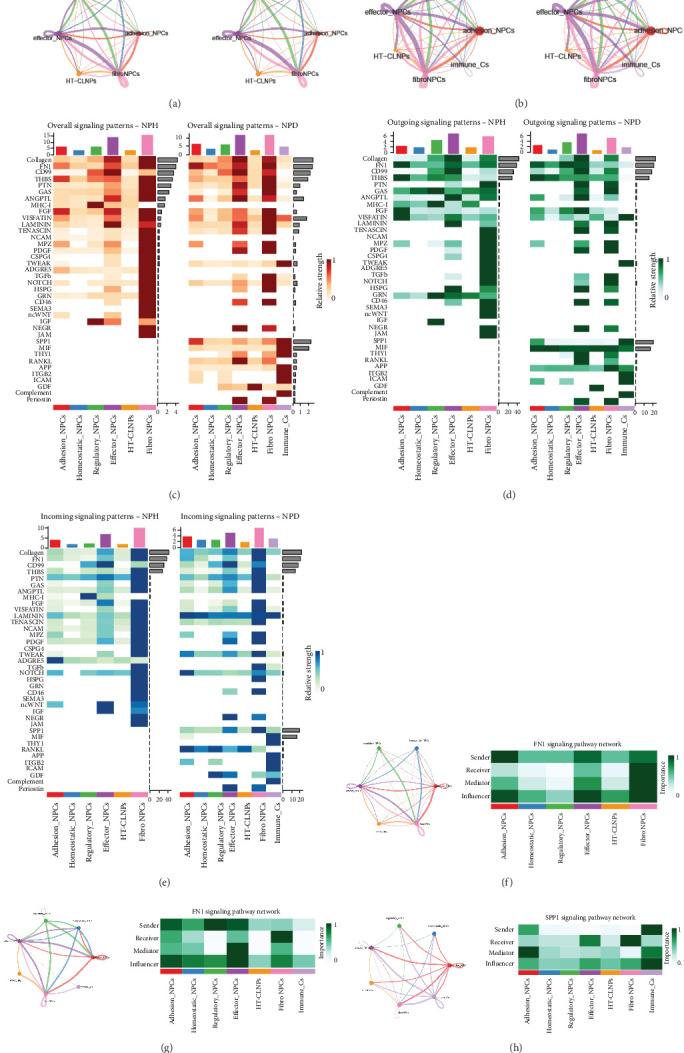
Cell–cell communication analysis of seven cell clusters in NP. (a) Number and strength of interactions between six cell clusters in health NP. (b) Number and strength of interactions between seven cell clusters in degenerated NP. (c–e) Heatmap showing cellular communication in the NPs before and after degeneration. (f) FN1 signaling pathway network in healthy NP. (g) FN1 signaling pathway network in degenerated NP. (h) SPP1 signaling pathway network in degenerated NP.

To determine alterations in intercellular ligand/receptor interactions during degeneration, we used the CellChat algorithm to probe cellular communication in 2 NP samples with different degrees of degeneration. Comparison of Figure 3a,b shows that intercellular communication was significantly increased after NP degeneration compared with that in healthy tissue; moreover, intercellular ligand/receptor interactions were altered, suggesting that immune cells infiltrating into the NP during degeneration have altered cellular communication compared with that in the original NP CellChat. Using weighted directed network measurements, we can separately identify the main senders of intercellular communication (senders), receivers (receivers), mediators (mediators), and influencers (influencers) of intercellular communication. The heatmap (Figures 3c–e) visually demonstrates the changes in cellular communication in the NPs before and after degeneration. The increased cellular communication after degeneration mostly involves immune cells functioning as senders or receivers. The main cellular communication pathways in healthy NPs were the COLAGEN, FN1, and CD99 signaling pathway networks, whereas the SPP1 signaling pathway network was added to the degenerated NPs. To investigate the changes in the main cellular communications before and after degeneration, we listed FN1 and SPP1 individually in healthy and degenerated NPs (Figures 3f–h). Owing to the addition of immune cells, the primary ligand emitters of the FN1 signaling pathway network changed from adhesion to regulatory NP cells, and fibro-NP cells, which are the primary mediator and effector pathways, changed to effector NP cells. Interaction did not change and remained as FN1-CD44 (Figure S2).

**Figure 4:**
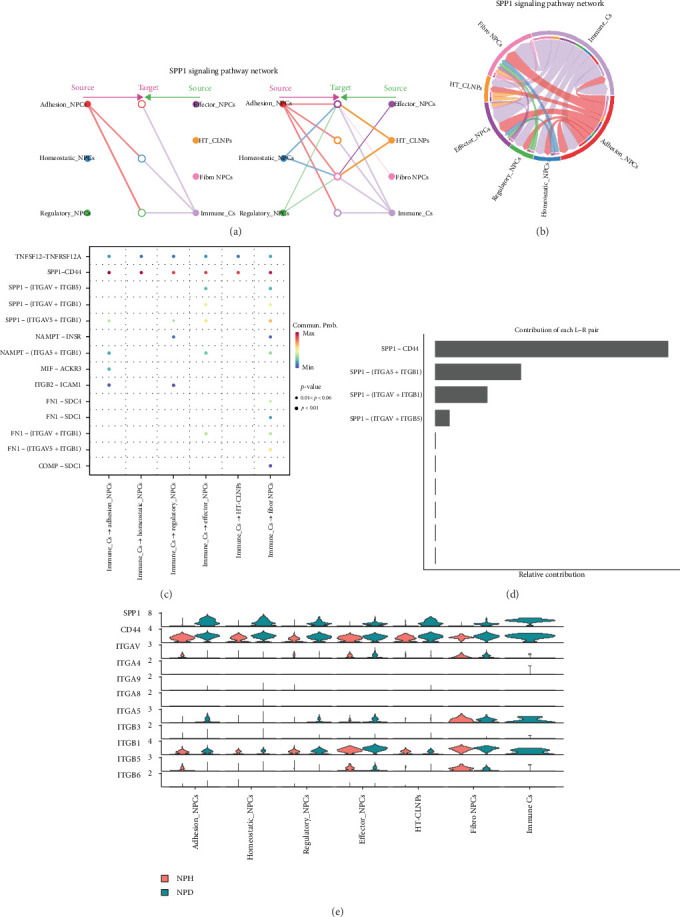
SPP1 signaling pathway network in degenerated NP. (a) Hierarchical plot showing SPP1 signaling pathway network. (b) Chord diagram showing SPP1 signaling pathway network. (c, d) Bubble plot and bar chart showing SPP1–CD44 is the most prominent ligand/receptor interaction in SPP1 signaling pathway network. (e) Violin plot showing major expressed genes in SPP1 signaling pathway network.

The SPP1 signaling pathway network is a major and unique intercellular communication pathway in degenerated NP. The SPP1 signaling pathway network ligands are mainly emitted by immune cells and act on the receptors of fibro-NP cells, whereas adhesion NP cells mainly play a delivery role in this pathway (Figure 4a,b). The SPP1 signaling pathway network consists of four major ligand/receptor interactions, the most prominent of which is SPP1-CD44 (Figure 4c,d). The major expressed genes in the SPP1 signaling pathway network were represented in each cell subtype using violin plot, which showed that SPP1 was mainly expressed in degenerated NPs, and the expression of the most prominent receptor, CD44, was also significantly higher in degenerated NPs than in healthy NPs (Figure 4e).

### 3.4. SPP1 Is Involved in IVDD Pathogenesis

**Figure 5:**
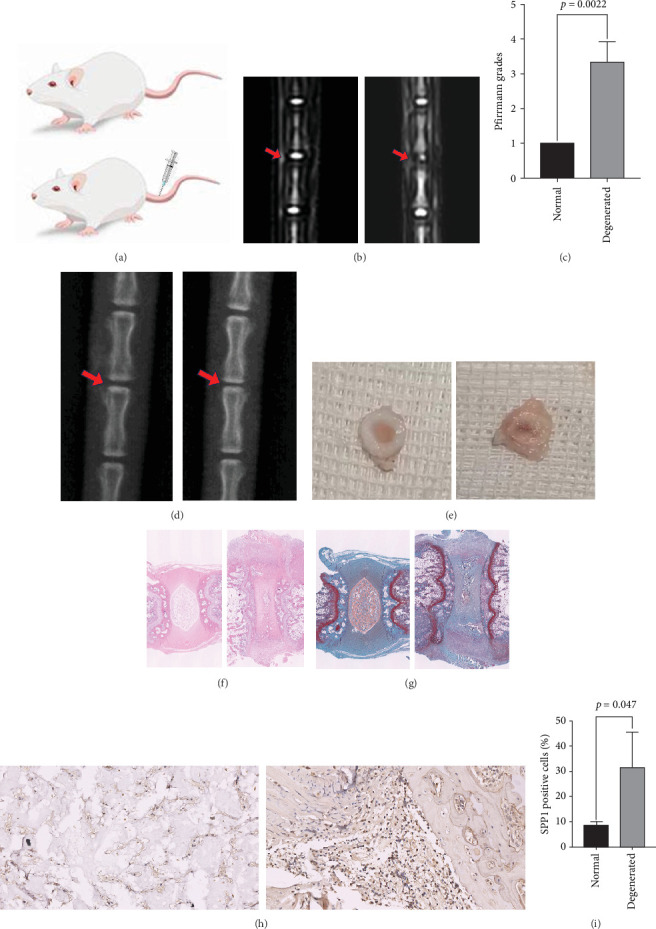
Expression of SPP1 in a rat model of caudal disc degeneration. (a) Establishment of an intervertebral disc degeneration model by needling the caudal intervertebral disc in rats. (b) The T2 weighted MRI shown are representative of the different groups 2 weeks after surgery. (c) Quantitative analysis of Pfirrmann grades. Data were presented as the mean ± SD, *n* = 3. *p*  < 0.05 (Student's *t* tests) (d) X-ray shown are representative of disc height changes 2 weeks after modeling. (e) Anatomical specimen display. (f) The pathological staining of rat intervertebral disc (hematoxylin and eosin staining, ×12.5). (g) The pathological staining of rat intervertebral disc (Safranin O/fast green staining, ×12.5). (h, i) IHC staining and quantitative analysis of SPP1 staining. Data were presented as the mean ± SD, *n* = 3. *p*  < 0.05 (Student's *t* tests).

Considering the high expression of SPP1-related genes in patients with IVDD, we established an IVDD model using rat caudal IVDs (Figure 5a) to validate our scRNA-seq results. The MRI confirmed that the IVDD model was successfully established (Figure 5b,c). X-ray showed a decrease in disc height after successful modeling (Figure 5d). Dissection of the discs showed that the NP of healthy discs was hydrated, full, and translucent, whereas that of the degenerated discs was significantly shrunken, mineralized, and hardened (Figure 5e). H&E and S-O staining were used to observe morphological changes in the IVDs. The results showed that the height of the IVDs, NP, and number of cells in IVDD rats were significantly reduced (Figure 5f). In addition, the red area decreased and the green area increased, indicating a decrease in proteoglycan content in rats with IVDD (Figure 5g), further confirming disc degeneration in our model rats. Immunohistochemical staining was performed to observe the expression of SPP1 in the healthy and degenerated intervertebral discs (Figure 5h). We found that SPP1 levels were significantly elevated in the NP tissues of IVDD rats (*p*  < 0.05) (Figure 5I). This suggests that SPP1 accumulates in NP tissues of patients with IVDD and may be responsible for disc degeneration.

**Figure 6:**
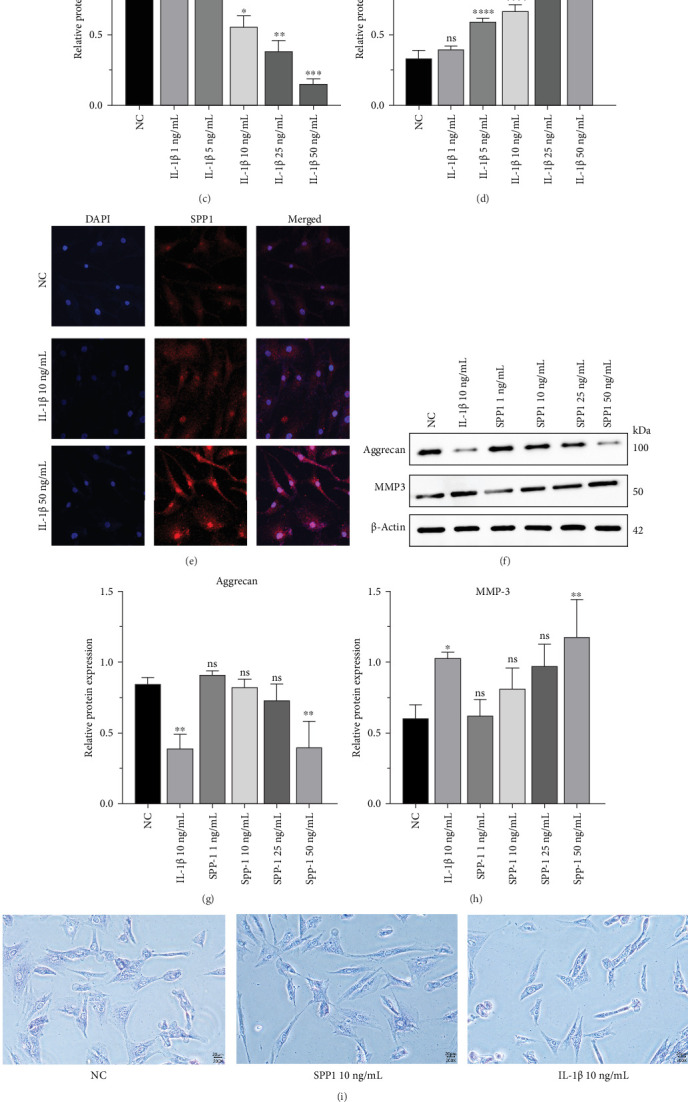
SPP1 is involved in and induces NP cell degeneration. (a) The mRNA expression of SPP1 gene was detected by qRT-PCR after treatment of NP cells with different concentrations of IL-1β. Data were presented as the mean ± SD, *n* = 3. ns, not significant; *⁣*^*∗*^*p*  < 0.05, *⁣*^*∗∗*^*p*  < 0.01, *⁣*^*∗∗∗*^*p*  < 0.001, *⁣*^*∗∗∗∗*^*p*  < 0.0001 (one-way ANOVA). (b–d) The protein level and quantifications analysis of Aggrecan and SPP1 in different group were measured by western blot. Data were presented as the mean ± SD, *n* = 3. ns, not significant; *⁣*^*∗*^*p*  < 0.05, *⁣*^*∗∗*^*p*  < 0.01, *⁣*^*∗∗∗*^*p*  < 0.001, *⁣*^*∗∗∗∗*^*p*  < 0.0001 (one-way ANOVA). (e) The colocalization of SPP1 (red) was examined by immunofluorescence in NP cells of NC group, IL-1β 10 ng/mL group, and IL-1β 50 ng/mL group. (f–h) The protein level and quantifications analysis of Aggrecan and MMP-3 in different group were measured by western blot. Data were presented as the mean ± SD, *n* = 3. ns, not significant; *⁣*^*∗*^*p*  < 0.05, *⁣*^*∗∗*^*p*  < 0.01, *⁣*^*∗∗∗*^*p*  < 0.001, *⁣*^*∗∗∗∗*^*p*  < 0.0001 (one-way ANOVA). (i) Toluidine blue staining was performed to observe morphological changes in NP cells across the NC group, SPP1 10 ng/mL group, and IL-1β 10 ng/mL group. (j) The colocalization of morphology was fluorescently stained with phalloidin in NP cells of NC group, SPP1 10 ng/mL group, and SPP1 50 ng/mL group. (k) The colocalization of Aggrecan (red) was examined by immunofluorescence in NP cells of NC group, IL-1β 10 ng/mL group, and SPP1 50 ng/mL group.

To further investigate the relationship between SPP1 and IVDD, we used IL-1β stimulation to construct an NP cell degeneration model to simulate the infiltration and entry of immune cells after IVDD and detected the expression of SPP1 and degeneration-related proteins by qrtPCR, western blotting, and IF. The results showed that SPP1 expression was significantly higher compared with that in normal NP cells after different concentrations of IL-1β treatment (Figure 6a). WB results showed that the expression of aggrecan gradually decreased with the increasing concentration of IL-1β, indicating that the degree of degeneration of NP cells increased with the increasing concentration of IL-1β (Figure 6b,c). The expressions of SPP1 and aggrecan were completely opposite; the SPP1 increased in an IL-1β concentration-dependent manner (Figure 6b,d). IF staining showed similar results. Using two representative concentrations of IL-1β (10 and 50 ng/mL) to treat NP cells, we detected the IF intensity of SPP1 expression, which was gradually increased, indicating that IL-1β significantly induced SPP1 expression in NP cells (Figure 6e).

To further explore the role of SPP1 in IVDD, we measured the protein expression of aggrecan and MMP-3 in NP cells treated with different concentrations of SPP1 for 24 h (Figures 6). With increasing concentrations of SPP1, the expression of aggrecan in NP cells gradually decreased and the expression of MMP-3 gradually increased, indicating that SPP1 significantly induced the degeneration of NP cells. The higher the concentration of SPP1, the more severe the degree of NP cell degeneration. The cells were fluorescently stained with toluidine blue and phalloidin to observe their morphological changes. Toluidine blue staining revealed that NP cells treated with SPP1 and IL-1β exhibited reduced cytoplasm and an elongated cellular morphology (Figure 6i). The results showed that compared with the control group, the fluorescence staining intensity of NP cells was lower after SPP1 stimulation, and the higher the concentration of SPP1, the greater the change in the morphology of NP cells, which were clearly elongated and exhibited a spindle shape (Figure 6j). Compared with the NC group, both IL-1β 10 ng/mL and SPP1 50 ng/mL led to a decrease in the expression of aggrecan fluorescence intensity in NP cells, indicating that both significantly induced the degeneration of NP cells (Figure 6k).

### 3.5. SPP1 May Be a Therapeutic Target to Reverse IVDD

**Figure 7:**
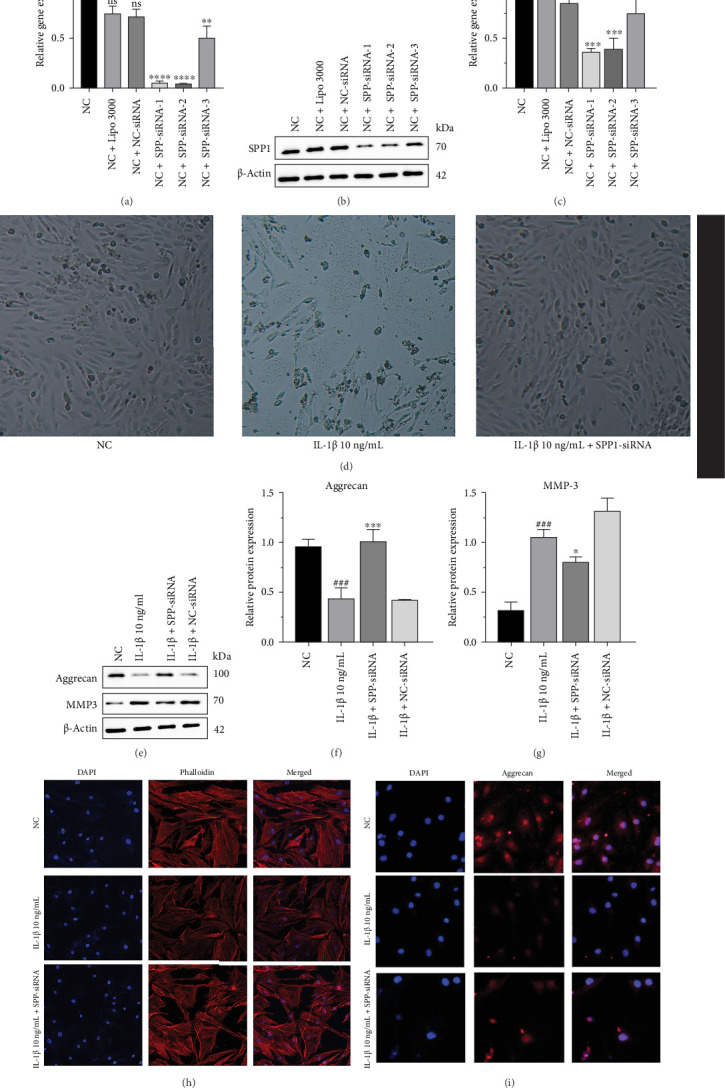
SPP1 may be a therapeutic target to reverse IVDD. (a) The mRNA expression of SPP1 gene was detected by qRT-PCR after transfection of NP cells with different siRNA. Data were presented as the mean ± SD, *n* = 3. ns, not significant; *⁣*^*∗*^*p*  < 0.05, *⁣*^*∗∗*^*p*  < 0.01, *⁣*^*∗∗∗*^*p*  < 0.001, *⁣*^*∗∗∗∗*^*p*  < 0.0001 (one-way ANOVA). (b, c) The protein level and quantifications analysis of SPP1 in different group were measured by western blot. Data were presented as the mean ± SD, *n* = 3. ns, not significant; *⁣*^*∗*^*p*  < 0.05, *⁣*^*∗∗*^*p*  < 0.01, *⁣*^*∗∗∗*^*p*  < 0.001, *⁣*^*∗∗∗∗*^*p*  < 0.0001 (one-way ANOVA). (d) The cell growth of each group was observed using an inverted microscope. (e–g) The protein level and quantifications analysis of Aggrecan and MMP-3 in different group were measured by western blot. Data were presented as the mean ± SD, *n* = 3. Compare to degeneration group: ns, not significant; *⁣*^*∗*^*p*  < 0.05, *⁣*^*∗∗*^*p*  < 0.01, *⁣*^*∗∗∗*^*p*  < 0.001. Compare to NC group: ns not significant; #*p* < 0.05, ##*p* < 0.01, ###*p* < 0.001, ####*p* < 0.0001. (one-way ANOVA). (h) The colocalization of morphology was fluorescently stained with phalloidin in NP cells of NC group, IL-1β 10 ng/mL group, and IL-1β 10 ng/mL + SPP1-siRNA group. (i) The colocalization of Aggrecan (Red) was examined by immunofluorescence in NP cells of NC group, IL-1β 10 ng/mL group, and IL-1β 10 ng/mL + SPP1-siRNA group.

Three different siRNAs were designed for screening and qRT-PCR was used to detect *SPP1* expression after transfection. The results showed that the expression of *SPP1* was significantly reduced after SPP1-siRNA-1 and SPP1-siRNA-2 transfection, indicating that SPP1-siRNA-1 and SPP1-siRNA-2 were the most efficient in transfection (Figure 7a). Western blot results (Figure 7b) show that all three SPP1-siRNAs significantly inhibited the expression of SPP1 protein after transfection of human NP cells and based on the statistical results (Figure 7c), SPP1-siRNA-1 was the most effective. Combined with the qRT-PCR results, SPP1-siRNA-1 was finally selected for transfection of human NP cells to construct SPP1-silenced human NP cells for subsequent experiments.

For the experiments, the cells were divided into four groups: control (normal myeloid cells, NC), degeneration (IL-1β 10 mg/mL), experimental (IL-1β 10 ng/mL + SPP1-siRNA), and experimental control group (IL-1β 10 ng/mL + NC-siRNA). After 24 h of treatment with IL-1β (10 ng/mL), the cell growth of each group was observed using an inverted microscope (Figure 7d). Compared with the control group, the number of cells in the degeneration group was significantly reduced and the cell morphology was more elongated. In the experimental group, the cell number and morphology were significantly better than those in the degeneration group (Figures 7). Due to SPP1 silencing in the experimental group, the expression of aggrecan protein was elevated compared with that in the degeneration group and the expression of MMP-3 protein was reduced. This indicates that inhibition of SPP1 expression can improve the degeneration of myeloid cells. Phalloidin staining showed that the fluorescence staining intensity of NP cells in the degeneration group was the lowest, whereas that of the experimental group rebounded (Figure 7h), which was similar to the WB results. The expression of aggrecan was elevated in NP cells after SPP1-siRNA transfection compared with the degeneration group (Figure 7i).

## 4. Discussion

IDDD is common; therefore, finding effective and innovative treatments for to prevent or reverse IVDD and improve the clinical outcomes of patients is important [27]. An in-depth understanding of the biological basis and pathophysiological processes underlying IVDD is required to develop new therapeutic strategies [28]. However, owing to its complex multifactorial processes and cellular heterogeneity, the internal homeostasis and microenvironment of the IVD have not been fully elucidated. The NP, as one of the most critical components of the IVD, has osmotic properties owing to its richness in COL2 and aggrecan, and maintains its height and compressive strength by retaining more fluids, which play a physiological function in the IVD [6]. Therefore, focused on the NP and explored NP changes during disc degeneration. We analyzed 2 NP samples with different degrees of degeneration donated by the same patient based on public databases and obtained 7633 cellular scRNA-seq data points after QC. Unlike previous studies that included different inter-individual NP samples, the present study depicted NP cell profiles more precisely by comparatively analyzing different NP samples from the same individual, excluding the effects of individual differences such as age, sex, and disease. According to the results of our analysis, NP tissues contained two main types of NP cells and immune cells, and further downregulation and identification of NP cells identified six cell subtypes, namely adhesion, homeostatic, regulatory, effector, HT-CLNP, and fibro-NP cells. TU et al. [14] reported a single-cell resolution transcriptional profile of human NP, which similarly identified six new human NP cell subtypes, which increases the credibility of the results of our analyses.

In our analytical results, the proportion of adhesion NP cells was close to 50% and was the predominant cell subtype in degenerated NP. Adhesion NP cells were mainly enriched in the TNFA_SIGNALING_VIA_ NFKB signaling pathway, suggesting that they produce inflammatory factors, such as TNF-α, during IVDD, and are perhaps the NP cell subset that is mainly involved in inflammatory responses in causing NP degeneration. In addition, the inflammatory response accelerates disc degeneration by affecting NP cell metabolism and the extracellular matrix microenvironment and causes discogenic pain by promoting abnormal nerve ingrowth into the disc [29]. Thus, adhesion NP cells may be the NP cell subtype most strongly associated with pain. Regulation of glycolysis is mediated by hypoxia-inducible factor-1 α (HIF-1α), a transcription factor that responds to local oxygen availability [30]. Adhesion NP cells in degenerated tissues were significantly enriched in the HIF-1 signaling pathway, suggesting that they are better adapted to the hypoxic environment than other NP cells. This explains why the number of adhesion NP cells increased rather than decreased with the number of degenerated cells in the disc. Fibro-NP cells, although relatively few, were the only other NP cell subtype with elevated cell numbers in degenerated NP. In pseudotime analysis, the trajectory differentiation of Fibro-NP cells showed two levels of differentiation, with a small portion located in the initiation segment, indicating that they may have some myeloid progenitor cell characteristics. This finding is similar to that of Tu et al. [14], who reported that CD90+ fibro-NP cells have myeloid progenitor cell characteristics. The NP progenitor cell properties of fibro-NP cells may explain why their cell numbers increased rather than decreased in degenerated NP.

Immune cells were only present in degenerated NP tissue, suggesting that immune cells were not originally present in the NP tissue but entered from outside the disc during degeneration. Ling et al. [31] revealed the important role of macrophages in human disc degeneration in an scRNA-seq analysis. The invasion of immune cells resulted in a marked enhancement of intercellular communication in NP tissues, adding a number of ligand/receptor signaling pathways that were not previously present, of which the SPP1 signaling pathway network was the most dominant. Based on cellular communication analysis [16], Zhou et al. proposed that SPP1 is a new clue in the microenvironment of IVDD, which is related to the occurrence and degradation of IVD.

SPP1, also known as osteobridging protein (OPN), is a transformation-associated phosphorylated protein found mainly in the extracellular matrix of mineralized tissues and in the extracellular fluid at sites of inflammation [32]. SPP1 binds to integrins and CD44, and plays a role in a variety of pathophysiological processes, including biomineralization, cellular immunity, inflammation, fibrosis, apoptosis, tumorigenesis, and metastasis [33]. Abnormal SPP1 expression is associated with a variety of skeletal disorders, and clinical studies have shown that serum SPP1 levels are positively correlated with the severity of osteoporosis and can be used as a biomarker for the early diagnosis of osteoporosis in postmenopausal women [34]. Many studies have found higher levels of SPP1 in the plasma and synovial fluid of patients with osteoarthritis than in healthy adults, indicating that SPP1 may be associated with the severity of osteoarthropathy. Studies on SPP1 in IVDD are still in their infancy. Gene profiling of human prominent IVD (H-IVD) and degenerative IVD (D-IVD) mesenchymal stem cells (MSCs) showed that D-IVD MSCs exhibited significant SPP1 overexpression compared with H-IVD-MSCs and that SPP1 expression appeared to be directly correlated with its Thompson classification, suggesting that SPP1 may be a potential marker of IVDD [35].

Based on analytical results, we observed immune cell infiltration and activation of the SPP1 signaling pathway in degenerated NP tissues, leading us to hypothesize that immune cells and SPP1 may be key factors mediating NP degeneration. By establishing a rat tail intervertebral disc needle-puncture model to simulate disc degeneration and employing immunohistochemical staining techniques, we confirmed significant upregulation of SPP1 expression in degenerated discs. This finding substantiates the critical role of SPP1 in the pathological process of IVDD. Subsequently, in vitro experiments were conducted by creating an inflammatory environment using IL-1β to simulate the internal milieu of IVDD. We observed the effects of inflammation on NPCs and found that SPP1 expression was upregulated under inflammatory stimulation. Integrated with single-cell data analysis results, this upregulation may originate from adhesion-NPCs responding to inflammatory stimuli. As an important non-collagenous matrix protein, SPP1 exhibits high binding affinity for Type I collagen and hydroxyapatite and has been shown to participate in the entire process of bone matrix mineralization [36, 37]. This characteristic of SPP1 may promote pathological mineralization within the NP tissue, ultimately leading to loss of physiological function and exacerbation of IVDD. To test this hypothesis, we treated NP cells with varying concentrations of SPP1 and evaluated degeneration-related markers compared with control groups. The results demonstrated that SPP1 induced NP degeneration in a concentration-dependent manner. These findings confirm that upregulated SPP1 expression can drive NP cell degeneration and identify SPP1 as a key molecular mediator in the initiation of NP degeneration.

Based on the results of previous studies, we hypothesized that during disc degeneration, immune cells invade the interior of the NP via inflammatory chemotaxis and neovascularization. Immune cells that enter the interior of the NP act on the NP cells by secreting SPP1, generating a series of chain reactions that ultimately accelerate NP degeneration. To verify the above hypothesis, we constructed SPP1-silenced human NP cells by transiently transfecting human NP cells with siRNA to interfere with their SPP1 expression and verified whether NP degeneration caused by IL-1β stimulation could be alleviated when SPP1 expression was reduced. In recent studies, SPP1 siRNA has been used to treat mice with rheumatoid arthritis, which resulted in a significant inhibition of synovial proliferation, leukocyte infiltration, and articular cartilage erosion. This indicated that SPP1 siRNA effectively mediated the depletion of SPP1, thereby inhibiting synovitis [38]. In our experiments, SPP1 siRNA effectively silenced the expression of SPP1 protein, and the degeneration of SPP1-silenced NP cells was significantly reduced in the experimental group compared with that in the degeneration group. This suggests that the inhibition of SPP1 expression ameliorated the degeneration of NP cells and that SPP1 may be a potential therapeutic target for blocking or reversing IVDD.

The present study has some limitations. First, the sample size of scRNA-seq data is relatively small and more healthy and degenerate samples are needed for comparative analyses in the future. Second, the NP cells were cultured on the surface of substrates rather than 3D culture, which cannot fully represent in vivo conditions. In addition, siRNA silencing of SPP1 for NP degeneration requires in vivo experimental validation.

## 5. Conclusion

Based on single-cell sequencing data, we revealed the changes in the NP cell atlas during IVDD and found that immune cells were only present in degenerated NP and that secreted SPP1 played a major role in cellular communication in degenerated NP. Thus, SPP1 may be a key molecule in NP degeneration. Subsequently, we experimentally verified that the expression of SPP1 was significantly elevated in a human NP degeneration model and caused the degeneration of human NP cells. Finally, silencing SPP1 expression using siRNA revealed that inhibition of SPP1 expression reversed the degeneration of NP cells. Our study suggests that SPP1 is a key molecule in the pathological process of IVDD, its expression level correlates with the severity of IVDD, and it may be a potential therapeutic target for blocking or reversing IVDD.

## Data Availability

The datasets generated and analyzed during the current study are available in the zenodo repository,https://zenodo.org/uploads/17070457, reference number DOI 10.5281/zenodo.17070457.

## Supporting Information

Supporting Information Process of calculations and experimental steps.
