# Supplementary material for: Changes in Nucleus Pulposus Cell Atlas and the Role of SPP1 During Intervertebral Disc Degeneration: Single-Cell Sequencing Analysis
Source: Mediators Inflamm. 2025 Nov 29;2025:5593429. doi: 10.1155/mi/5593429 (PMC12681403; doi:10.1155/mi/5593429)
Supplement: Supporting Information — Process of calculations and experimental steps. [file 5593429.f1.docx]

**Fig.S1 A**

After quality control and doublet exclusion filtering to remove cells with low gene detection (< 200 genes) and high mitochondrial gene content (> 20%).

**Fig.S1 B**

The variance diagram shows the variation of gene expression in all cells of NP. The red dots represent highly variable genes and the black dots represent non-variable genes.

**Fig.S1 C**

Classification of cell clusters in each sample.

**Fig.S1 D**

Expression levels of NP marker genes for each cell cluster.

**Fig.S2 A**

Bubble plot showing FN1-CD44 is the most prominent ligand/receptor interaction in FN1 signaling pathway network.

**Fig.S2 B**

Bar chart showing FN1-CD44 is the most prominent ligand/receptor interaction in FN1 signaling pathway network in healthy NP.

**Fig.S2 C**

Bar chart showing FN1-CD44 is the most prominent ligand/receptor interaction in FN1 signaling pathway network in degenerated NP.
